# Commentary: The Ross reversal: A rare operation to be done by experts only

**DOI:** 10.1016/j.xjtc.2021.02.003

**Published:** 2021-02-04

**Authors:** Emile Bacha

**Affiliations:** Division of Cardiothoracic Surgery, Department of Surgery, Columbia University Irving Medical Center–New York Presbyterian Hospital, New York, NY

**Figure:**
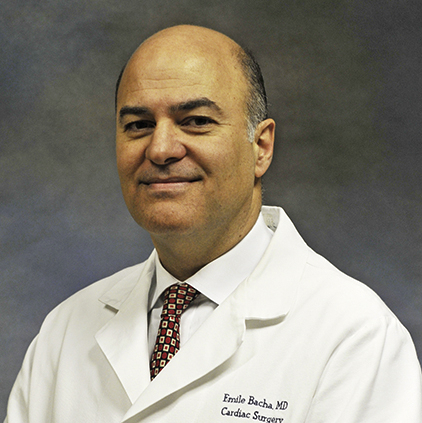
Emile Bacha, MD

**Central Message**

The Ross Reversal can be an effective procedure for the right patient. Deciding when to do it in the face of only theoretical benefits can be difficult.

See Article page 417.

Weiss and Peterson^1^ should be congratulated on a very nice and complete technical description of the Ross reversal, an operation designed to maximally use available native tissues by placing the (failed) autograft back in the pulmonary position with the hope of avoiding issues related to a prosthetic right-sided conduit. The authors' extensive experience becomes abundantly clear when reading the text. Technical pitfalls, such as injury to the left main coronary while dissecting the proximal homograft, keeping a nice rim on the coronary button (not always easy to do), and detaching the autograft from the left ventricular outflow tract (LVOT), are lucidly described. There are really only 2 technical aspects in which I would differ from the authors. First, I prefer to complete the right-sided implantation before starting the left side, just for convenience and ease of exposure's sake. Second, in very dilated autografts, I have found it necessary to downsize and trim the dilated autograft from a balloon-shaped structure to a tube-shaped one with a simple linear running suture line in the middle of the sinus to avoid having something that looks like a pulmonary artery aneurysm in the left upper mediastinum.

Having said all that, the issue is more about when this operation should be done and who should do it. Clearly, any operation for Ross failure, be it a valve-sparing procedure or an aortic root replacement, is an exercise of high-level technical virtuosity coupled with intense judgment calls. Deciding whether an autograft (neo-) aortic valve is worth saving in the systemic circulation is a complex judgment that involves both clinical, such as the patient's age and overall health, and anatomic criteria, such as the sizes of the aortoventricular junction and the sinotubular junction, the tissue quality, the shape and sizes of the cusps, and others. Therefore, adding one more layer to that judgment call, namely whether to excise the autograft in toto and call it “good enough” for the right side while potentially simultaneously weakening the tissues at the distal LVOT site where the proximal suture line stiches are going to sit is not a judgment call that the “occasional” aortic root surgeon should be making. The putative advantages of the Ross reversal are simply not clear enough.
